# Decision Rules and Group Rationality: Cognitive Gain or Standstill?

**DOI:** 10.1371/journal.pone.0056454

**Published:** 2013-02-22

**Authors:** Petru Lucian Curşeu, Rob J. G. Jansen, Maryse M. H. Chappin

**Affiliations:** 1 Department of Organization Studies & Center for Innovation Research, Tilburg University, Tilburg, The Netherlands; 2 Department of Organization Studies, Tilburg University, Tilburg, The Netherlands; Arizona State University, United States of America

## Abstract

Recent research in group cognition points towards the existence of collective cognitive competencies that transcend individual group members’ cognitive competencies. Since rationality is a key cognitive competence for group decision making, and group cognition emerges from the coordination of individual cognition during social interactions, this study tests the extent to which collaborative and consultative decision rules impact the emergence of group rationality. Using a set of decision tasks adapted from the heuristics and biases literature, we evaluate rationality as the extent to which individual choices are aligned with a normative ideal. We further operationalize group rationality as cognitive synergy (the extent to which collective rationality exceeds average or best individual rationality in the group), and we test the effect of collaborative and consultative decision rules in a sample of 176 groups. Our results show that the collaborative decision rule has superior synergic effects as compared to the consultative decision rule. The ninety one groups working in a collaborative fashion made more rational choices (above and beyond the average rationality of their members) than the eighty five groups working in a consultative fashion. Moreover, the groups using a collaborative decision rule were closer to the rationality of their best member than groups using consultative decision rules. Nevertheless, on average groups did not outperformed their best member. Therefore, our results reveal how decision rules prescribing interpersonal interactions impact on the emergence of collective cognitive competencies. They also open potential venues for further research on the emergence of collective rationality in human decision-making groups.

## Introduction

Small social groups are information processing systems [1], [2]. As such, cognitive science concepts and models have been extensively used to explore the ways in which groups perform cognitive tasks, such as decision making and problem solving. Recent empirical evidence points toward the existence of collective intelligence, or the “c” factor that explains collective performance of groups in a variety of cognitive tasks [3]. Woolley and colleagues argue that collective intelligence is a group property that transcends individual cognitive competencies and describes the group as a whole [3]. Woodley and Bell [4] criticized the concept of collective intelligence and argued that the procedure used to arrive at the concept would rather reflect a common overarching factor of collective cognitive performance, similar to the General Factor of Personality (GFP). In other words, the “c” factor could be, in fact, a group level-manifestation of the pro-social orientations (high cooperativeness and low contentiousness) associated with the GFP [4]. Either way, whether an emergent group property or group level manifestation of GFP, a central issue related to collective cognitive competencies is the extent to which they are open to manipulation and change. In their concluding comments, Woolley and colleagues call for research that elucidates the extent to which collective intelligence or, more generally, collective cognitive competencies can be increased via specific group interventions [3].

Curşeu and Schruijer [5] report a series of three studies in which they show that simple normative interventions that stimulate collaboration and participation foster group cognitive complexity, increase group rationality, and improve decision quality. Their second study, however, only compared decision-making groups that received norms for achieving consensus with groups that did not receive consensus norms [5]. As groups without normative interventions could (in principle) use various decision rules, it is important to better understand the way in which specific normative interventions induce specific forms of interpersonal interactions and ultimately influence the emergence of collective rationality. Interpersonal interactions in decision-making groups are often regulated through decision rules that prescribe the ways in which individual contributions are to be combined into collective outputs [6], [7]. Two rules that have received substantial attention in the literature on group decision making [6], [8], [9] are consultative (one member collects inputs from the other group members and makes the choice) and collaborative (all members provide informational input used to achieve consensus on the collective choice). Consequently, the aim of this study is to test the extent to which the use of these two group decision rules impacts on the emergence of group rationality.

### Collective Cognitive Competencies as Cognitive Synergy

The group cognition literature to date has mostly focused on structural views of cognition and explored ways in which shared individual representations impact on collective performance [1]. Groups draw on the cognitive resources of their members and, therefore, have larger cognitive capacities than individuals alone [10], yet little to no attention is shown to the emergence of collective competencies in (human) decision-making groups [5]. We use the framework advanced by Hackman [11], and further developed by Larson [12], [13], to argue that collective cognitive competencies reflect the synergetic cognitive processes in groups. Effective synergetic processes occur when group performance (collaborative performance) exceeds the performance achieved by the simple, preprogrammed combination of standalone group members’ efforts [12], [13]. Hackman [11] defines group synergy as a group level phenomenon that emerges from the interactions among members and affects how well a group deals with task-related demands and opportunities [11]. Larson [12], [13] further refined the concept and defined group synergy as an objective gain in group performance as compared to summed individual performances that is attributable to interpersonal interaction and collaboration. Larson [12], [13] also differentiates between strong and weak group synergy. Groups achieve weak synergy when collective performance is better than the average performance of group members, and strong synergy when collective performance exceeds the performance of the best performing individual in the group [13]. We use this framework to describe emergent cognitive competencies (in particular rationality) as group level (collective) information processing competencies that emerge from interpersonal interactions and transcend individual cognitive competencies.

Rationality has received considerable attention in the decision-making literature and two major views on rationality currently co-exist. On the one hand, “classical” rationality is related to a set of cognitive competencies for decision-making that concur with a normative ideal [14], and on the other hand, ecological rationality explores the extent to which decisions are adapted to the environment in which decision makers operate [15], [16]. Consequently, two operationalizations of rational behavior exist. According to the first one, decisions are rational to the extent to which they conform to the norms of logic and mathematics [14], and rational behavior is evaluated based on the principles of consistency and coherence. According to the second one, ecologically rational behavior is the result of adaptation and it reflects accurate judgments about facts in the surrounding environment [16].

In this paper we build on the “classic view” [14] of rationality and define it as reduced sensitivity to decision-making biases and heuristics and the general capability of making logically correct choices [14]. Research on cognitive heuristics and biases has identified various instances in which decision makers deviate from a normative ideal and, as such, sensitivity to heuristics and biases offers a reliable framework to evaluate individual and group rationality [5], [17]. Building on the intra-individual consistency principle, Parker and Fischhoff [18] introduced the Adult Decision-Making Competence measure. Lack of sensitivity to biases (answer consistency across various items) is used to evaluate decision makers’ rationality and a decision maker is considered to be rational if his/her answers adhere to the consistency principles [18]. Decision makers can, in principle, be non-contradictory in their preferences, but this does not mean their choices are logically correct. In order to capture rationality as conformity to logic, statistics and probability principles, we use a modified set of decision tasks adapted from the general literature on heuristics and biases. The decision-making tasks are modified in such a way that participants have the chance to select from a multiple answer set the normatively correct answer to the decision situation [5].

In terms of achieving synergy, if collective rationality in a decision-making task exceeds the average group members’ rationality, the group achieves weak cognitive synergy. Strong cognitive synergy reflects the objective gain in collective rationality above and beyond the best (most rational) group member. Group rationality is therefore, conceptualized as a group emergent property (collective cognitive competence) generated by the coordination of individual cognitive competencies (i.e., individual rationality) during social interactions [5]. Research on decision making in non-human groups has extensively addressed the emergence of collective rationality [10], [19], [20] and has shown that interactions among individual group members help the group make rational choices, even if the individual group members do not fully explore all available alternatives. In other words, although composed of “irrational” members, animal groups can behave rationally [6], [19], [20]. Collective rationality emerges from the complex pattern of behaviors and interactions among individual members, and it reflects a set of collective cognitive competencies evolved from selection processes that operate at the group level [20]. As opposed to human decision-making groups where feedback on choices (and their possible consequences) is delayed and often ambiguous, animal groups receive timely and unambiguous feedback in situations with high adaptive value (often directly linked to the survival of the group). It is therefore reasonable to argue that the emergence of collective cognitive competencies in human decision-making groups is explained by qualitatively different mechanisms than the ones operating in animal colonies. In line with the arguments of emergent cognition, group rationality is expected to be influenced by the rationality of individual members and the interaction processes induced by the group decision rules [5].

### Decision Rules and Rationality

Decision rules prescribe interpersonal interactions and influence information sharing and integration during decision making. Consultative and collaborative decision rules are among the most common decision rules described in the group decision-making literature and extensively used in (human) organizations [8], and also documented in animal groups [6], [19].

In consultative decisions, the group follows the decision of the formal leader, and the role of the group members is simply to provide informational input to the central decision maker. Consultative decisions are argued to be more efficient, yet information integration is rather limited as central decision makers seem to prefer informational inputs that are similar or support their own view and disregard those that diverge from their own view [9]. Moreover, appointed leaders may overuse their power, dominate the group, and ultimately reduce group communication and participation [21]. We expect, therefore, that the potential for information integration, and ultimately the emergence of collective cognitive competencies, is impeded by the use of a consultative decision rule. In collaborative settings, the group as a whole makes the decision through consensus and members have equal participation rights during the decision-making process. Such decentralized decision making is conducive to the emergence of rational behavior in ant colonies [19] and simulation studies show that stable social groups benefit from collaborative rather than follow-the-best decision rules [6]. To conclude, because members have the chance to openly discuss and contribute with their unique knowledge and expertise to decision making, collaborative settings are expected to be conducive for the emergence of group rationality that ultimately transcends the rationality of individual group members. Previous research supports this argument and shows that group norms that stimulate collaboration and participation create group synergy that ultimately leads to the emergence of complex collective cognitive structures, better decision quality, and higher group rationality [5]. Therefore, as collaborative decision rules have more potential for knowledge sharing and integration, we hypothesize that collaborative decision rules are superior to consultative decision rules in generating group rationality.

## Methods

### Ethics Statement

Participants were asked to participate in a group decision exercise as part of their course related activities, and they were informed that the aim of the exercise is to illustrate the use of various decision rules in group decision making. Verbal consent was asked before the beginning of the class, and participants were informed that their results would be used in scientific research. All participants gave their verbal consent to participate in the study, and because the decision exercise was part of regular curricular activities, no supplementary consent was asked from the local ethics committee. The main task involved filling out an individual and a group questionnaire that did not involve any personal data with the potential of embarrassing the participants. The study was carried out in The Netherlands and according to the local ethical guidelines, studies based on questionnaires that do not require any personal data with the potential to embarrass the participants are exempted from ethical committee approval. The experiment was organized as a participative learning exercise (part of the coursework) and no foreseeable risks, beyond those present in routine daily life, were anticipated in this study. Nevertheless, participants were informed that if they experienced distress associated with their participation in the exercise, they should notify the teachers immediately. All participants were debriefed after the experiment as part of the reflection on the participative learning exercise.

### Participants and Procedure

Six-hundred-seventeen first year students enrolled in an introductory course at a Dutch University with an average age of 19.15 years, 369 female and 248 male, participated in the study. Participants were informed that they would take part in a participative learning exercise aimed at illustrating the role of decision rules on decision outcomes. The participative learning exercise was part of regular curricular activities in the first course related to organization studies in their curriculum, and organizational decision making was one of the topics taught in the course. During the workshop, all participants first performed a set of decision tasks. Then they were randomly assigned to 176 small groups (average group size 3.49) and asked to redo the same decision tasks as a group. We used two sets of instructions to manipulate the decision rule. The ninety one groups in the collaborative condition received the instruction to approach the task by employing the method of group consensus, which means that each group member must agree on the alternative that will be selected by the group. In order to reach consensus in the collaborative condition, group members were allowed to discuss their opinions and views on the decision tasks with each other. The eighty five groups in the consultative condition received the instruction to approach the task by employing the method of group leader, which means that each group member must provide input to the appointed group leader who then decides on the alternative that will be selected on behalf of the group. In this condition, members were not allowed to discuss with other group members, and the group leader was randomly appointed by the teacher. After completing the group task, participants compared the average individual scores with the collective score and were debriefed about the study. The details of the manipulation were also presented so students could reflect on the group dynamics induced by the use of the two group decision rules. Along with this debriefing, the results of the decision task were used to discuss implications for group composition choices for different types of organizations, as well as the implications for organizational decision making. Data were collected across three academic years in the same course, and in order to stick to the learning goals of the workshops, both experimental conditions were used in each workshop (students in each workshop were organized in small groups having 3 to 4 members, and approximately half the groups were placed in the consensus and half in the consultative decision rule). Researchers kept a logbook in which all details of the study across the three years were fully recorded.

### Measures

*Decision rationality* was evaluated using ten decision-making tasks adapted from the most frequently used experimental procedures in the decision-making heuristics and biases literature, namely the framing effect (2 items), representativeness bias (6 items), and Ellsberg’s paradox (2 items). The items were adapted in such a way that the normative correct alternative was presented among these alternatives and, as a consequence, it is possible to compute a rationality score reflecting the extent to which individual choices deviate from a normative ideal. The following example illustrates the way in which the classic example of the Asian Disease problem [22] used to elicit framing effects was modeled in our rationality measure: “Imagine that your country is preparing for the outbreak of an unusual Asian disease, which is expected to kill 600 people. Two alternative programs to combat the disease have been proposed: Program A and Program B. Assuming that the exact scientific estimates of the consequences of the programs are known, which one will you choose as the most effective? (a) If Program A is adopted, 200 people will be saved; (b) If Program B is adopted, there is a ⅓ probability that 600 people will be saved, and ⅔ probability that no people will be saved; (c) Both programs are equally effective and (d) I cannot decide.” An adapted item for representativeness is: “You have the chance of buying a lottery ticket. Suppose that on the first ticket the numbers are 7, 12, 18, 24, 33 and 45 and on the second ticket, the numbers listed are 1, 2, 3, 4, 5 and 6. Which one do you think has the highest chance of being winner?(a) The first ticket; (b) The second ticket; (c) Both tickets have equal chances of being a winner; (d)I cannot decide” and for Ellsberg’s paradox: “Suppose you have an urn with 90 balls, 30 yellow and 60 red or blue. You can draw one ball from the urn and you have to bet on the color of the ball. If you correctly guess the color of the ball, you can earn 100$. Which color do you think has the highest probability of being drawn? (a)Yellow; (b) Red; (c) Both have equal probability of being drawn; (d) I cannot decide”.

For each decision task, the normative correct answer (i.e. answer “c” in the examples above) was rated with one point while the other (selected) answers received zero points, and the total score for the rationality in decision making is computed by adding the partial item scores. Low scores are indicative for decision makers being sensitive towards decision-making biases and heuristics, while high scores indicate a lower sensitivity to these biases and heuristics. This way of evaluating rationality is aligned with the classic conceptualization of rationality [14], and previous research shows that rationality scores correlate positively with the self reported rational decision-making style [23]. Because we have evaluated individual rationality as well as group rationality with the same instrument, we can compute both weak as well as strong synergy [13]. Weak cognitive synergy was computed by subtracting the average individual rationality score from group rationality (the results of the group decision task), while strong group synergy was computed by subtracting the highest individual score from the group score.

Given that group size and group diversity are important for collective rationality [6], [7], we used group size, average age (computed as the average age of group members), age diversity (computed as within group age standard deviation), and gender diversity (computed using the Teachman index of diversity) as control variables in our analyses [24]. The selection of control variables is based on previous research, showing that group size is likely to impact on group coordination and information exchange [25]. Moreover, average within group age and gender diversity influence interpersonal interaction and communication [26], and gender diversity is also related to the pattern of interpersonal interactions and the emergence of collective knowledge structures [1].

## Results

Means, standard deviation and correlation for the variables included in the study are presented in Table 1.

**Table 1:** Correlation Table with Descriptive Statistics (N = 176).

	Mean	SD	1	2	3	4	5	6
1. Group size	3.49	.68	1					
2. Age mean	19.15	1.37	.391**					
3. Age SD	1.16	1.04	.203**	.709**				
4. Gender diversity a	.36	.31	.191*	.000	.009			
5. Average IR	4.38	1.11	.160*	.193*	.193*	-.002		
6. IR SD	1.44	.73	.067	.131	.091	.040	.268**	
7. Group rationality	5.13	1.98	.108	.114	.096	.125	.712**	.294**

Notes.

* p<.05;

** p<.01;

a 0 = male, 1 = female; SD – standard deviation; IR – individual rationality; numbered columns represent the variables specified on respective rows.

To test our hypothesis, we ran two OLS regression analyses with weak and strong cognitive synergy as dependent variables. As controls, we used group size, average age, age and gender diversity, as well as within group rationality mean and standard deviation. We supplemented the regression with a bootstrapping procedure to compute the 95% confidence intervals for the effect sizes. The results of the regression analyses and the 95% confidence intervals are presented in Table 2.

**Table 2:** Results of the OLS Regression Analyses for Weak and Strong Cognitive Synergy (N = 176).

	Weak cognitive synergy		Strong cognitive synergy	
	B(SE)	95%BCaCI	B(SE)	95%BCaCI
*Control variables^a^*				
Group size	−.074 (.16)	[−.42;.28]	−.465 (.15)***	[−.84; −.06]
Age mean	.007 (.11)	[−.20;.22]	.020 (.19)	[−.19;.25]
Age SD	−.067 (.13)	[−.33;.20]	−.040 (.18)	[−.30;.22]
Gender diversity b	.817 (.32)**	[.12; 1.54]	.926 (.43)***	[.21; 1.66]
Average individual rationality	.222 (.09)*	[.03;.40]	.237 (.14)**	[.05;.41]
Individual rationality SD	.283 (.14)*	[.001;.54]	−.825 (.13)***	[−1.19; −.54]
*Main effect manipulation*				
Experimental condition c	.736 (.20)***	[.34; 1.12]	.730 (.20)***	[.28; 1.18]
R_sq_	.16		.26	
F change step 2	13.39***		12.82***	

a† p<.10;

* p<.05;

** p<.01;

*** p<.001,

b 0 = male, 1 = female,

c 0 = consultative, 1 = collaborative; BCaCI – bias corrected accelerated confidence intervals.

**Figure 1:**
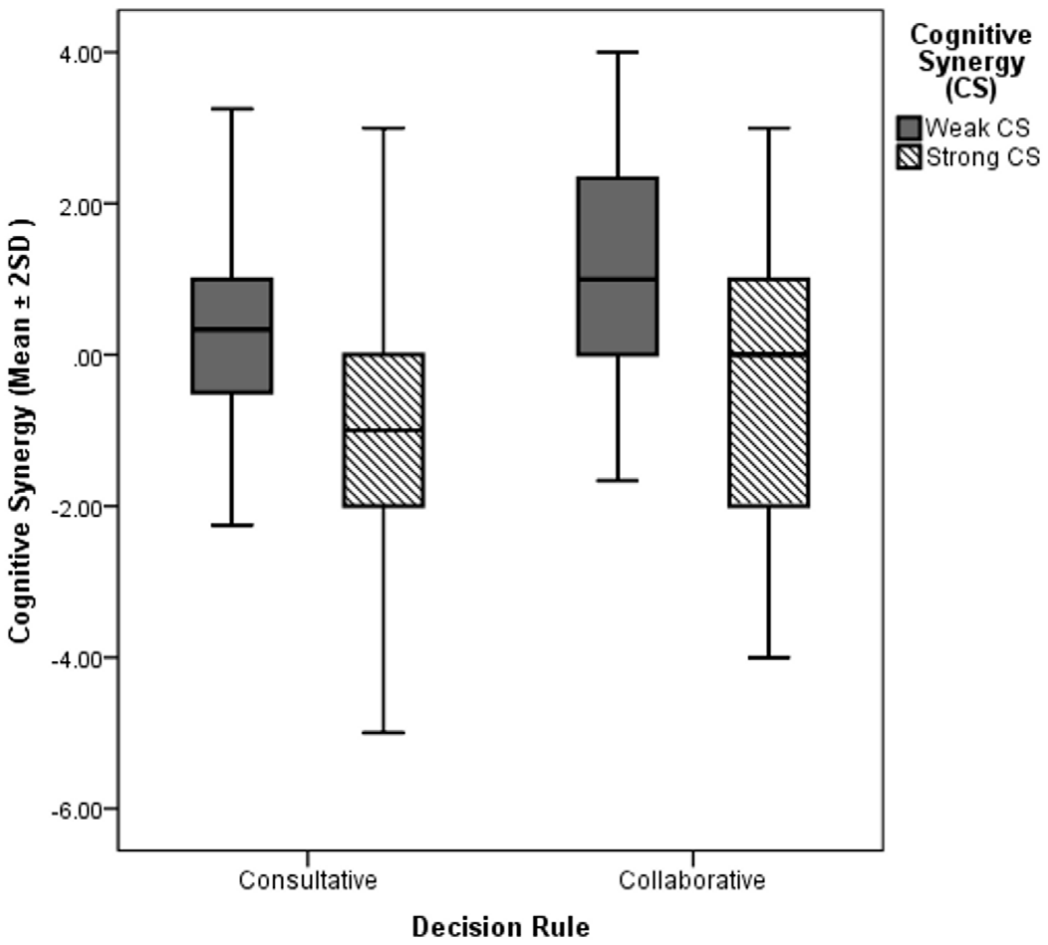
Weak and strong cognitive synergy in consultative and collaborative decision making conditions.

As the results indicate, the effect of the experimental manipulation is significant in both regression analyses, and the 95% confidence interval does not include zero. Therefore the hypothesis that collaborative decision rules are superior to consultative decision rules in fostering group rationality is fully supported. Weak cognitive synergy is also positively and significantly predicted by the within group standard deviation of the individual rationality score and gender diversity. As the mean score was used in the formula of weak cognitive synergy, a plausible explanation for this positive effect is the association between the mean and standard deviation [24]. Strong cognitive synergy has also a negative association with the within group standard deviation of individual rationality, as well as with the group size. Similar to Woolley and colleagues [3], our results show that gender diversity has a positive and significant association with both strong and weak cognitive synergy. Figure 1 depicts the mean scores (±2SD) for weak and strong cognitive synergy for the two experimental conditions. As shown in Figure 1, average strong cognitive synergy values are negative for both experimental conditions, indicating that on average the highest individual rationality score in the group exceeds the group rationality score.

## Discussion

Our results support the hypothesis that the collaborative decision rule is superior to the consultative one in generating both weak and strong cognitive synergy (operationalized as collective rationality) in group decision making. These results have three important implications for the literature on group decision making. First, we extend the concept of rationality from individuals to groups and show that groups have information processing capabilities that transcend the individual capabilities of their members. We challenge the view that groups accentuate their members’ sensitivity to heuristics and biases, and show that collaborative decision rules have the potential of creating weak cognitive synergy. Our results are in line with insights from animal decision making showing that complex information integration in ant colonies prevents collective irrational behaviors [20]. Second, we show that decision rules have the potential to influence the emergence of these collective cognitive competencies. We, therefore, add to the literature on collective cognitive competencies [5], [3] and show that decision rules that guide within group interaction processes have the potential of influencing the emergence of group cognitive competencies. Third, we distinguish between weak and strong cognitive synergy, and we show that although collaborative decision rules increase both strong and weak cognitive synergy, in absolute terms they only generate weak cognitive synergy. Because average strong synergy has negative values in both experimental conditions, we can conclude that although the groups that worked using the collaborative decision rule achieve higher levels of strong synergy, group rationality is actually lower than the highest individual rationality within the group. This observation is aligned with simulation results of decision-making in animal groups [6] showing that in single-shot decisions, experts systematically outperform groups.

An important question arises from these results, namely how can groups develop strong synergy in its absolute sense? An indicative answer could be offered by the study of Watson, Michaelsen and Walt [27], showing that the group consensus method repeatedly used over time stimulates strong synergy in groups. Given the centrality of team development processes in these results, the evolution of meta-cognitive processes (transactive memory systems, cross-understanding and group meta-cognition) may play an important role in the emergence of strong cognitive synergy and the development of collective cognitive competencies. Repeated interpersonal interactions facilitate meta-cognitive processes (the way in which individual members reflect on how the group performs a cognitive task), and thus generate more opportunities for interpersonal and cognitive synergies in groups. As transactive memory systems develop (shared awareness of who knows what in the group), groups become more efficient in first identifying and then using the specific resources of their best member [6]. Therefore, future research should explore the emergence and development of collective cognitive competencies using longitudinal perspectives.

Group meta-cognition in decision making refers to shared individual cognition about the way groups make decisions (the way individual members think about the way groups process information and decide [28]) and are likely to impact on interpersonal interaction and directed information search, which ultimately enhance cognitive synergy in decision-making groups. Group meta-cognitive processes could also shed more light on how groups achieve strong synergy through the adjustment of the complex behavioral algorithm that drives information integration in social aggregates [20]. Future research could, for example, explore the extent to which meta-cognition influences information sharing in groups and the adjustment of individual communication behavior in an attempt to achieve high quality decisions.

Cross-understanding is yet another possible path towards achieving strong cognitive synergy in groups, as it reflects the extent to which group members have accurate representations of each other’s knowledge, skills and expertise [29]. Enhanced cross-understanding could eventually increase the reliance on the most knowledgeable group member and could also help the group to overcome the detrimental effects of fragmentation. As indicated by our results, within group standard deviation of individual rationality (high standard deviation is indicative of group fragmentation, see Harrison and Klein [24]) has a negative impact on strong cognitive synergy. Cross-understanding might help groups with high fragmentation (half of members scoring high on rationality and half scoring low) better integrate subgroups and, as a consequence, may stimulate strong synergy to emerge. Cross-understanding is, therefore, one of the conditions under which diversity in cognitive competencies trumps cognitive ability effects in group decision-making (7).

Along with its contributions, our study also has several limitations. First, the scoring procedure for the task used to evaluate rationality could have been a boundary condition for evaluating strong cognitive synergy in our study. In particular, groups in which the best performing individual scored a ten on the individual decision task (performed all decision tasks correctly) could not achieve strong cognitive synergy. We performed a robustness check and excluded from our analyses the groups that had this particular configuration. The results of our analyses did not change and therefore we can conclude that our interpretations are accurate. Second, we adopted a particular view of rationality (as conformity to a normative ideal) and did not explore the emergence of ecological rationality. Reimer and Katsikopoulos [30] explored the less-is-more heuristics in decision-making groups and their results challenged the common assumption that groups make better decisions when they have more information (also supported by our collaborative decision rule), and they show that the less-is-more heuristic holds in groups using recognition based majority rule. It becomes highly relevant to further explore the specific processes through which collective ecological rationality emerges in (human) decision-making groups. Third and finally, our results reflect the superiority of the collaborative decision rule, yet we did not explicitly address the way interpersonal interactions (i.e., social network structure) influence the emergence of collective rationality. According to simulation studies [31], [32] interpersonal interaction is a key element for the emergence of collective rationality. Therefore, future research should tap into the relationship between communication structure and emergence of collective cognition.

### Conclusions

The results presented in this paper provide initial empirical evidence for the effect of decision rules on group rationality. We show that the collaborative decision making context is conducive to the emergence of group rationality, conceptualized as a collective cognitive competence of making choices aligned with a normative ideal. Weak and strong cognitive synergy are used to operationalize group rationality as the gain in rationality (over the average within group individual rationality and the most rational member of the group) attributed to interpersonal interactions induced by decision rules. As rationality of choice is an important asset in managerial decisions, our results have important implications for the management of decision- making groups in organizations.
